# Supplementary material for: Parents’ views of psychological research with children: Barriers, benefits, personality, and psychopathology
Source: PLoS One. 2023 Jun 23;18(6):e0287339. doi: 10.1371/journal.pone.0287339 (PMC10289465; doi:10.1371/journal.pone.0287339)
Supplement: S1 Table — Note. N = 109. Italic font indicates corresponding factor. 1all correlations p ≤ .001. (DOCX) [file pone.0287339.s001.docx]

S1 Table. *Parents‘ Barriers for Participating in Research - Questionnaire (P-BARQ): Items, factor loadings, mean values (M, scale: 1–5), standard deviations (SD), and item-total correlations (r_it_)*

| No. | Item | Factor 1 | Factor 2 | *M* | *SD* | *r*_it_^1^ |
| --- | --- | --- | --- | --- | --- | --- |
| 1 | I am unsure what to expect when we participate. | .11 | *.19* | 3.25 | 0.95 | .31 |
| 2 | I cannot find time to participate. | *.81* | -.09 | 3.09 | 1.09 | .44 |
| 3 | My child has no time to participate. | *.77* | <.01 | 2.72 | 1.04 | .50 |
| 4 | I do not want my child or me to disclose personal information. | .07 | *.86* | 3.15 | 1.17 | .74 |
| 5 | The effort for a participation is usually too high. | *.53* | .14 | 3.04 | 1.10 | .50 |
| 6 | I am not interested in scientific research. | .17 | *.27* | 1.92 | 0.95 | .36 |
| 7 | My child has no interest in scientific research. | -.09 | *.35* | 2.99 | 1.13 | .35 |
| 8 | I am thinking about what negative effects participation could have for my child. | -.10 | *.62* | 3.31 | 1.35 | .55 |
| 9 | The expense allowance for participation is usually too low. | .26 | *.44* | 2.85 | 0.99 | .58 |
| 10 | Most topics do not affect us. | .35 | *.56* | 2.85 | 0.99 | .71 |
| 11 | I do not want others to know personal information about our family. | -.14 | *.91* | 3.25 | 1.29 | .68 |
| 12 | I have little knowledge about current scientific research projects in which my child could participate (i.e. little information about where which studies are offered). | .06 | *.15* | 4.04 | 1.11 | .33 |
| 13 | I do not think my child's participation will make a significant contribution to scientific insight. | .26 | *.38* | 2.64 | 1.17 | .51 |
| 14 | I am not sure whether the information on confidentiality and data protection will be adhered to as stated. | <.01 | *.65* | 2.64 | 1.36 | .61 |
| 15 | The dates for participation are not flexible enough (e.g. not compatible with everyday school life). | *.35* | .34 | 2.89 | 0.95 | .55 |

*Note.* *N* =109. Italic font indicates corresponding factor. ^1^all correlations *p* ≤ .001
