# Supplementary material for: Parents’ views of psychological research with children: Barriers, benefits, personality, and psychopathology
Source: PLoS One. 2023 Jun 23;18(6):e0287339. doi: 10.1371/journal.pone.0287339 (PMC10289465; doi:10.1371/journal.pone.0287339)
Supplement: S2 Table — Note. N = 109. Italic font indicates corresponding factor. 1all correlations p ≤ .001. (DOCX) [file pone.0287339.s002.docx]

S2 Table. *Parents‘ Benefits for Participating in Research - Questionnaire (P-BERQ): Items, factor loadings, mean values (M; scale 1–5), standard deviations (SD), and item-total correlations (r_it_)*

| No. | Item | Factor 1 | Factor 2 | *M* | *SD* | *r*_it_^1^ |
| --- | --- | --- | --- | --- | --- | --- |
|  | What advantages should a scientific study have for you or how important are the points mentioned below to you? …. |  |  |  |  |  |
| 1 | ... the duration of the participation is as short as possible  (i.e. under one hour). | *.55* | -.34 | 4.01 | 0.91 | .37 |
| 2 | ... the participation takes place exclusively online (from home). | *.57* | -.40 | 4.06 | 0.95 | .38 |
| 3 | … a telephone call with the head of studies or investigator  is made before participation. | *.24* | .07 | 3.10 | 1.03 | .30 |
| 4 | … there is a sufficient expense allowance (i.e. approx. 10 €/h). | *.58* | .01 | 3.46 | 1.02 | .59 |
| 5 | ... the travel costs can be covered independently of an expense  allowance. | *.60* | .13 | 3.79 | 1.01 | .63 |
| 6 | … is explained, how my child's participation could help other  children. | *.90* | -.12 | 4.28 | 0.80 | .63 |
| 7 | … I/my child can learn more about psychological research or  scientific work. | *.69* | -.04 | 3.84 | 0.94 | .57 |
| 8 | … I/we would receive feedback on our individual results. | *.76* | .26 | 4.11 | 0.92 | .70 |
| 9 | … I/we will be informed about the group results (i.e. over all  participants). | *.49* | .06 | 3.93 | 0.94 | .44 |
| 10 | ... a diagnostic interview is conducted and I receive  feedback about my child's mental health. | .55 | *.67* | 3.70 | 1.18 | .74 |
| 11 | .... I/we receive feedback as to whether or to what extent  my child has a psychological problem or disorder. | .59 | *.68* | 3.72 | 1.20 | .77 |
| 12 | ... an intelligence diagnosis is carried out on my child and  I receive feedback on this. | .34 | *.65* | 3.57 | 1.18 | .69 |
| 13 | … I could talk to a psychologist about possible problems at  home. | -.03 | *.75* | 3.28 | 1.04 | .43 |
| 14 | … in order to get advice on education or tips on how to deal  with problems. | <.01 | *.83* | 3.37 | 1.09 | .46 |

*Note. N* = 109. Italic font indicates corresponding factor. ^1^all correlations *p* ≤ .001
