# Supplementary material for: Parents’ views of psychological research with children: Barriers, benefits, personality, and psychopathology
Source: PLoS One. 2023 Jun 23;18(6):e0287339. doi: 10.1371/journal.pone.0287339 (PMC10289465; doi:10.1371/journal.pone.0287339)
Supplement: S3 Table — Note. N = 109. (DOCX) [file pone.0287339.s003.docx]

S3 Table. *Items on previous study participation and willingness to participation with mean values (M) and standard deviations (SD)*

| Items | *M* | *SD* |
| --- | --- | --- |
| How often have you participated in psychological studies so far? | 4.13 | 21.60 |
| How often has/have your child(ren) participated in psychological studies (in agreement with you) so far? | 0.09 | 0.87 |
| How would you currently rate your willingness to participate in a psychological study? (1–5) | 3.55 | 0.91 |
| How would you currently rate your willingness to support your child's participation in a psychological study? (1–5) | 2.91 | 1.10 |
| How would you currently rate your child's willingness to participate in a psychological study (1–5) | 2.66 | 1.10 |

*Note.* *N* =109
