# Supplementary material for: Parents’ views of psychological research with children: Barriers, benefits, personality, and psychopathology
Source: PLoS One. 2023 Jun 23;18(6):e0287339. doi: 10.1371/journal.pone.0287339 (PMC10289465; doi:10.1371/journal.pone.0287339)
Supplement: S4 Table — Note. N = 109. (DOCX) [file pone.0287339.s004.docx]

S4 Table. *Six different study titles with mean values (M) and standard deviations (SD) (willingness participating, scale: 1–5)*

| Title | *M* | *SD* |
| --- | --- | --- |
| Research project seeks children and adolescents as participants | 2.47 | 1.18 |
| Study of the University XY looks for children and adolescents as participants | 3.04 | 1.15 |
| Scientific study seeks children and adolescents as participants | 2.85 | 1.10 |
| Research project seeks young participants between the ages of xx and xx | 2.99 | 1.17 |
| Study of the University XY seeks young participants aged between xx and xx years | 3.45 | 1.22 |
| Scientific study seeks young participants aged between xx and xx years | 3.28 | 1.17 |

*Note.* *N* =109
